# Attention Deficit-Hyperactivity Disorder Symptoms and Daytime Voiding Symptoms in Children with Primary Enuresis: An Observational Study to Evaluate the Effectiveness of Desmopressin Treatment

**DOI:** 10.1155/2015/356121

**Published:** 2015-03-17

**Authors:** Teng-Kai Yang, Ya-Jun Guo, Hong-Chiang Chang, Hung-Ju Yang, Kuo-How Huang

**Affiliations:** ^1^Division of Urology, Department of Surgery, Yonghe Cardinal Tien Hospital, New Taipei City, Taiwan; ^2^College of Medicine, Fu Jen Catholic University, New Taipei City, Taiwan; ^3^Department of Psychiatrics, Taipei City Hospital, Heping Branch, Taipei, Taiwan; ^4^Department of Urology, National Taiwan University Hospital, Taipei, Taiwan

## Abstract

*Purpose*. To evaluate the effectiveness of presence of desmopressin in treating primary enuresis (PE) for children with attention deficit-hyperactivity disorder (ADHD) symptoms. *Materials and Methods*. Children aged from 5 to 12 years with the chief complaint of PE treated with desmopressin were enrolled in pediatric urology clinics. The parent-reported SNAP-IV questionnaire was used to evaluate ADHD symptoms (cut-off value: 90th percentile). Voiding symptoms were assessed by the Dysfunctional Voiding Scoring System (DVSS) questionnaire. The responses to desmopressin were analyzed in children with and without ADHD symptoms. *Results*. The study sample comprised 68 children; 27 (39.7%) presented with ADHD symptoms and 41 (60.3%) with non-ADHD symptoms. The children collected from a tertiary referral center may explain the high prevalence of ADHD symptoms in the present study. The total DVSS score in the ADHD symptoms group was significantly higher than in the non-ADHD symptoms group (7.72 versus 5.65, *P* = 0.05). In the ADHD symptoms group, there were significantly higher score in the “pee 1-2 times/day” and “can't wait” subscales of DVSS and lower sleep quality based on the Pediatric Sleep Quality questionnaire, as well as significantly lower peak flow rate and voided volume. The responses to desmopressin for enuresis were comparable between children with ADHD and non-ADHD symptoms. *Conclusions*. Approximately 39.7% of PE children presented with ADHD symptoms at urologic clinics. PE children with ADHD symptoms had higher risk of daytime LUTS and comparable response to desmopressin treatment for PE. To evaluate ADHD symptoms and daytime voiding symptoms is important in children with PE.

## 1. Introduction

Pediatric enuresis or nocturnal incontinence defined by International Children's Continence Society [1] is a common, distressing condition which may bring personal inconvenience and social stress to children and their family [2]. A history of childhood, primary enuresis was more likely to lead to adult urgent incontinence or mixed incontinence [3]. Enuresis may link both psychological and emotional disorders [4, 5]. Several studies have demonstrated a significantly increased prevalence of ADHD in enuretic children [6–8]. One recent study also indicated the high prevalence of ADHD symptoms (42%) in children with lower urinary tract symptoms/enuresis in a tertiary referral center [9].

Desmopressin is one of the two standard treatments for primary enuresis (PE), the other being the alarm [10], but the evidence-based therapy about efficacy for enuresis in ADHD children is scarce. Only one study conducted combination therapy with desmopressin and oxybutynin, which showed a fair result for ADHD children with enuresis [11]. So we perform a prospective study to investigate the effectiveness of desmopressin alone for PE in children with ADHD symptoms.

## 2. Materials and Methods

### 2.1. Study Design and Subjects

The study protocol was approved by the Institutional Research Ethics (no. 200907060R). Between September 2010 and August 2011, we consecutively collected children aged from 5 to 12 years whose chief complaint was PE (wetting the bed three or more times/week) for more than one month at the urology clinics of the National Taiwan University Pediatric Hospital. Subjects were excluded if they had (1) concurrent neurologic or urogenital disorders, (2) congenital disorders, (3) prior previous pelvic surgery, or (4) concurrent use of medications which are known to interfere with bladder or sphincter function. All study subjects received bladder and renal ultrasonography and urinalysis to exclude other genitourinary tract anomalies and urinary tract infection. All enrollee first received 4-week bladder training program at the beginning of the treatment protocol, including evenly distributed fluid intake throughout the day, the avoidance of drinks precipitating bladder overactivity, timed voiding every 2-3 hours, fluid restriction in the evening, and emptying bladder before bedtime. For subjects with good compliance, if enuresis did not demonstrate significant improvement, we proceeded with medical therapy. We administered 12-week desmopressin orally, starting with a standard dose of (0.2 mg) and increasing after 2 weeks to 0.4 mg in subjects if the episodes of enuresis did not become completely dry. We attempted to wean the subjects from the medication since 10th week by administration of one-half of the dose for two weeks before discontinuation. Patients were followed up 1 month after cessation of medications.

The validated Chinese language version of the SNAP-IV was completed by the parent(s) of each enrollee [12–14]. The SNAP-IV includes 3 subscales, with items relating to inattention (items 1 to 9), hyperactivity/impulsivity (10 to 18), and oppositional defiant disorder (19 to 26). The SNAP-IV is based on a rating scale of 0 to 3 for each item, with 0 indicating not at all, 1 just a little, 2 quite a bit, and 3 very much. Scores for each of the 3 subscales were calculated by summing the scores of the items within each subscale and then dividing by the number of items in each subscale. Study subjects were classified as having ADHD symptoms if their combined score for the first 2 subscales was at or above the 90 percentile level based on norms for the Chinese language SNAP-IV. All other subjects (those below the 90 percentile level) were classified as non-ADHD. It is noteworthy that ADHD symptoms did not actually qualify for ADHD diagnosis, which is not merely made by completing the SNAP questionnaire.

Each subject, together with parents, answered the 10 questions of the validated Chinese language version of the Dysfunctional Voiding Symptom Scale (DVSS) questionnaire, which is commonly used to evaluate LUTS in children [15, 16]. The first 9 questions of the DVSS assess daytime incontinence, enuresis, constipation, urgency, voiding frequency, and dysuria, each scored on a scale of 0 to 3, with 0 indicating never or almost never, 1 less than half the time, 2 about half the time, and 3 almost every time. Question 10 assesses recent high stress events within the family. Scores were compared for each question individually and in the aggregate. In addition, the validated, Chinese version of Pediatric Sleep Quality (PSQ) questionnaire was used to assess the sleep quality of the children [17]. Constipation was defined as a delay or difficulty in defecation, present for two or more weeks and sufficient to cause significant distress to the patient [18].

The demographic data were elicited by a specially trained interviewer. Uroflowmetric studies were then conducted. The subjects were instructed to drink water until a strong desire to void was achieved, and they were then instructed to void into a uroflowmetry.

During the whole course of treatment, the numbers of wet nights per week were recorded. The number of wet nights during the 2 weeks before desmopressin treatment and the last 2 weeks at 4th month follow-up was compared to evaluate the effectiveness of desmopressin treatment. Nonresponse was defined as a 0–49% decrease, partial/complete response as more than 50% decrease in the number of wet nights [19].

### 2.2. Statistical Analysis

The numerical data were described by means and standard deviations (SD). The categorical data were expressed as counts and percentages. The numerical data were compared with Student's *t*-test, paired *t*-test, or nonparametric test. The chi-square or Fisher's exact test was used to compare the categorical data. Two-sided tests were used, and a *P* value less than 0.05 was considered to be statistically significant. All data in the present study were analyzed with commercial statistical software (SPSS version 13.0 for Windows, SPSS Inc., Chicago, IL).

## 3. Results

A total of sixty-eight children (51 boys and 17 girls) aged 5 to 12 years were enrolled; 27 (39.7%) presented with ADHD symptoms and 41 (60.3%) with non-ADHD symptoms, based on norm of the Chinese SNAP-IV parents form. Demographic data of the subjects are listed in Table 1. There were no significant differences between ADHD symptoms and non-ADHD symptoms group in terms of age, parental age, birth history, maternal history, and household income.

The daytime LUTS and peak flow rate (*Q*_max⁡_) and voided volume in two groups are shown in Table 2. The mean total DVSS score in children of ADHD symptoms group was significantly higher than in subjects in non-ADHD symptoms group (8.44 ± 5.63 versus 5.66 ± 3.95, *P* = 0.019). In terms of mean scores of all DVSS subscales, the items “I only go to the bathroom one or two times each day” and “I cannot wait when I have to pee” were significantly higher in the ADHD symptoms group (*P* = 0.031 and 0.019, resp.). ADHD symptoms children suffered more from initial urgency than non-ADHD symptoms children (25.9% versus 4.9%, *P* = 0.012, data not shown).

The *Q*_max⁡_ in uroflowmetry (UFM) was significantly lower in ADHD symptoms group (13.5 ± 5.97 versus 17.1 ± 7.26, *P* = 0.047) as well as lower voided volume (69.9 ± 43.2 versus 112 ± 75.1, *P* = 0.018). The sleep quality of ADHD symptoms group was significantly worse compared to non-ADHD symptoms group (total sleep quality score, 3.07 ± 1.88 versus 2.05 ± 1.26, *P* = 0.017). There was no difference in constipation between the two groups.

The responses to desmopressin for enuresis were shown in Table 3. Compared to non-ADHD symptom subjects, ADHD symptom subjects had comparable and favorable response rate (75.6% versus 66.7%, *P* = 0.42). The responses between non-ADHD symptom subjects and ADHD subtypes were comparable as well (versus inattention, *P* = 0.45; versus hyperactivity-impulsivity, *P* = 0.31; versus combined type, *P* = 0.86). No serious adverse effect was encountered for both groups during treatment.

Table 4 showed the results that three questionnaire scores of ADHD symptoms subjects, as well as *Q*_max⁡_ and voided volume, were all comparable between partial/full response and poor response groups (all *P* > 0.05).

## 4. Discussion

Most pediatricians and pediatric urologists have mentioned that ADHD children disproportionately suffered from various forms of voiding issues, such as enuresis, urgent incontinence, and dysfunctional voiding [6–8, 20]. In this study, we found that ADHD symptoms occurred in surprisingly high percentage (39.7%) of children with PE. Baeyens et al. stated in a study with similar result that parents rate children higher on ADHD scales due to the frustrating consequences of the voiding problem [21]. Our hospital being a tertiary referral center might explain the disproportionately high prevalence of neuropsychiatric symptoms in the present study. In other words, the material is probably biased towards children with complicated enuresis, since they sought help at a tertiary clinic. Nevertheless, the present study is the first to investigate the differential effectiveness of desmopressin in treating children with PE in terms of the presence of ADHD symptoms. Secondary enuresis can be a sign of an underlying medical or emotional problem. The etiology could be much more complicated, including urinary tract infection and structural or neurological problems. Moreover, the treatment algorism for secondary enuresis could be much more complicated. Therefore, we excluded subjects with secondary enuresis from the study.

Desmopressin and alarm are standard treatment for primary monosymptomatic enuresis. Desmopressin alone accounts for 40 to 81% success rate and effectively reduces voids at night during treatment [10, 22]. Nevertheless, the success rate of desmopressin treatment for enuresis varied in previous studies due to different study design and inconsistent definitions on success rate. In our study, partial and complete responses to desmopressin in children of different groups were within the previously reported range. Additionally, our results showed that the response rates were all comparable between non-ADHD and three ADHD subtypes; however, PE children with combined type experienced the most favorable response rate (73.3%). Since ADHD symptoms group suffered from significantly lower functional bladder capacity (voided volume), which is an independent predictor for response to desmopressin [23], the failure rate was expected higher than non-ADHD symptoms group. The potential predictors for response to desmopressin were evaluated as well, but no independent predictor was found in the present study.

Chertin et al. combined desmopressin and oxybutynin for enuresis in ADHD subjects and showed significant improvement in DVSS compared to that in the imipramine group, and the incidence of enuresis was significantly decreased as well [11]. It had to be mentioned that these children diagnosed with ADHD had more severe voiding symptoms than our patients with ADHD symptoms (mean DVSS 20.5 versus 8.44), and this showed that desmopressin alone had feasible effects for the subjects in the present study.

The correlations of enuresis with ADHD symptoms have not been investigated in children visiting pediatric urologic clinics, where the prevalence of ADHD diagnosed by the SNAP-IV MTA rating scale was high (39.7%) in the present study. Previous studies have found a higher prevalence of ADHD symptoms in enuretic children and linked the relationship between ADHD and voiding dysfunction [7, 24, 25]. Comorbid ADHD with enuresis may be related to central dopaminergic neurochemical dysfunction [26], and one previous study indicated that the atomoxetine, an approved ADHD medication, significantly increased dry nights number among enuretic children [27].

In the present study, the percentage of urgency as the initial presentation and the items “I only go to the bathroom one or two times each day” (infrequent voiding) and “I cannot wait when I have to pee” (urgency) were significantly higher in the ADHD symptoms group, similar to results from other series [6, 8]. This result reflects that ADHD symptoms children will need more education about voiding habits and may suffer more from stressful urgency that will result in lower self-esteem if urgent incontinence occurred [28].

Common sleep problems were related to ADHD such as shorter sleep duration, difficulties getting up, and more daytime sleep and account for behavior difficulties [29]. Sleep restriction leads to negative impact on the neurobehavioral functioning of ADHD subjects [30]. In the present study, based on the Pediatric Sleep Quality questionnaire, there was significantly worsened total sleep quality score in ADHD symptoms group (3.07 ± 1.88 versus 2.05 ± 1.26, *P* = 0.017), which should have been evaluated and treated in pediatric urology clinics. Some of the deficits in attention and hyperactivity may be alleviated after treatment [31].

There are some limitations in the present study. First, the number of study subjects is limited. Since subjects of non-ADHD symptoms seem to have more favorable response compared to those of ADHD symptoms, further studies with larger sample size are warranted to clarify the result. Second, the relapse of enuresis and the coexisted daytime voiding symptoms is not evaluated after desmopressin treatment. As previously reported, the relapse rate after cessation of treatment is high (30–50%) and relapse might follow within a short period of time [32]. Third, ADHD symptoms and uroflow in this study were assessed in only one setting. Repeated measurements may be necessary to increase the accuracy and obtain the precise information [33]. Moreover, children with ADHD symptoms in this study did not actually qualify for ADHD diagnosis. The diagnosis of ADHD requires multimodal (diagnostic interviews and questionnaires) and multi-informant (parents and teachers) assessment. At last, although there were no major adverse events and no drop-out during follow-up, detailed numbers of adverse events were not reported in the present study.

## 5. Conclusions

The prevalence of ADHD symptoms is high in children with PE at urologic clinics. Enuretic children with ADHD symptoms were more likely to have daytime LUTS. Desmopressin was effective in treating PE children regardless of the presence of ADHD symptoms. Further investigations are required to clarify the detailed voiding patterns in children with ADHD symptoms and long-term desmopressin response to PE.

## Figures and Tables

**Table 1:** Demographic characteristics categorized by the presence of ADHD symptoms in a total of 68 children with primary enuresis in urologic clinics.

	ADHD symptoms (*n* = 27)	Non-ADHD (*n* = 41)	*P* value
Age (yrs) mean ± SD	7.92 ± 2.05	8.58 ± 1.84	0.17
Paternal age (yrs) Mean ± SD	41.4 ± 6.66	42.9 ± 4.78	0.32
Maternal age (yrs) Mean ± SD	38.3 ± 5.16	40.4 ± 4.36	0.07
Birth weight (gm) Mean ± SD	3210 ± 412	3130 ± 560	0.56
Number of siblings (person)	1.19 ± 0.68	1.05 ± 0.59	0.38
Gender f/m (%)	8/19 (29.6/70.4)	9/32 (22.0/78.0)	0.47
Gestation age (%)			0.45
Full term	22 (81.5)	33 (80.5)	
Premature (<37 wks)	3 (11.1)	2 (4.9)	
Postterm (>40 wks)	2 (7.4)	6 (14.6)	
Maternal education (%)			0.26
College	8 (29.6)	17 (41.5)	
High school	19 (70.4)	22 (53.7)	
Elementary school	0 (0)	2 (4.8)	
Household incomes (%)			0.99
Low	3 (11.5)	5 (12.5)	
Middle	10 (38.5)	15 (37.5)	
High	13 (50.0)	20 (50.0)	
Marital status (%)			0.19
Intact marriage	23 (85.2)	38 (95.0)	
Separated	2 (7.4)	0 (0)	
Divorced	2 (7.4)	2 (5.0)	
Type of delivery (%)			0.80
Normal spontaneous delivery	20 (74.1)	28 (68.3)	
Emergent cesarean section	2 (7.4)	5 (12.2)	
Elective cesarean section	5 (18.5)	8 (19.5)	
Perinatal insult (%)	2 (8.3)	2 (5.3)	0.63

Household incomes: low means monthly incomes <30000 NT dollars; moderate means incomes between 30000 and 80000 NT dollars; high means incomes ≧80000 NT dollars.

Numeric data are expressed as mean ± SD and compared with *t*-test.

Categorical data are expressed as number (percentage) and compared with chi-square test.

**Table 2:** LUTS and flow rates in study subjects categorized by ADHD symptoms.

	ADHD symptoms (*n* = 27)	Non-ADHD (*n* = 41)	*P* value
	Mean ± SD	Mean ± SD	
Total DVSS	8.44 ± 5.63	5.66 ± 3.95	0.019
Subscale			
Wet underwear	0.52 ± 0.75	0.51 ± 0.84	0.97
Soak underwear	0.96 ± 1.09	0.66 ± 1.02	0.24
No daily bowel movement	1.04 ± 0.81	0.83 ± 0.95	0.35
Push to have bowel movement	0.93 ± 0.92	0.63 ± 0.92	0.20
Pee 1-2 times/day	0.59 ± 0.97	0.20 ± 0.51	0.031
Hold pee	1.33 ± 1.14	1.15 ± 0.91	0.74
Can't wait	1.56 ± 1.34	0.90 ± 1.07	0.019
Push to pee	0.15 ± 0.60	0.07 ± 0.26	0.48
Hurt when pee	0.07 ± 0.27	0.05 ± 0.22	0.67
Stressful events	1.33 ± 1.52	0.66 ± 1.26	0.06
Peak flow rate, mL/s	13.5 ± 5.97	17.1 ± 7.26	0.047
Voided volume, mL	69.9 ± 43.2	112 ± 75.1	0.018
Total sleep quality score	3.07 ± 1.88	2.05 ± 1.26	0.017
Constipation pt. number (%)	13 (48.1)	20 (48.8%)	0.96

Numeric data are expressed as mean and SD and compared with *t*-test.

Categorical data are expressed as number (percentage) and compared with chi-square test.

**Table 3:** The response of desmopressin for total subjects categorized by ADHD type (all *P* > 0.05 compared to non-ADHD).

	Non-ADHD	ADHD symptoms		
		Inattention	Hyperactivity/impulsivity	Combined
Pt. number	41	5	7	15
Partial/complete response	30 (75.6%)	3 (60%)	4 (57.1%)	11 (73.3%)
Nonresponse	11 (24.4%)	2 (40%)	3 (42.9%)	4 (26.7%)

Categorical data are expressed as number (percentage) and compared with chi-square test.

**Table 4:** Clinical measurements of 27 subjects with ADHD symptoms categorized by the response to desmopressin.

	Partial/complete response (*n* = 18)	Nonresponse (*n* = 9)	*P* value
	Mean ± SD	Mean ± SD	
SNAP sum	44.4 ± 14.0	46.6 ± 13.1	0.82
Total DVSS	7.58 ± 4.55	11.0 ± 7.25	0.11
Total sleep quality score	2.89 ± 1.73	3.56 ± 2.13	0.40
Peak flow rate, mL/s	16.3 ± 6.19	12.7 ± 5.77	0.17
Voided volume, mL	82.0 ± 46.3	67.2 ± 42.2	0.34

Numeric data are expressed as mean and SD and compared with Mann-Whitney test.

Categorical data are expressed as number (percentage) and compared with chi-square test.

## Abbreviations

ADHD: Attention deficit-hyperactivity disorder
DVSS: Dysfunctional voiding scoring system
LUTS: Lower urinary tract symptoms
PE: Primary enuresis
PSQ: Pediatric Sleep Quality
*Q*_max⁡_: Peak flow rate.
